# Kinetic study of rare earth elements extraction from decrepitated magnet powder using liquid magnesium

**DOI:** 10.1039/d3ra05796h

**Published:** 2023-11-07

**Authors:** Nicolas Stankovic, Julien Jourdan, Jérôme Marin, Alexandre Chagnes, Thibault Quatravaux

**Affiliations:** a University of Lorraine, CNRS, GeoRessources F-54000 Nancy France; b University of Lorraine, CNRS, Institut Jean Lamour F-54000 Nancy France thibault.quatravaux@univ-lorraine.fr

## Abstract

This study presents a comprehensive investigation of neodymium extraction from decrepitated magnet powder using liquid magnesium. Neodymium extraction from the decrepitated magnet into the liquid magnesium was assessed between 700 and 900 °C by measuring the average length of the diffusion zone in sintered samples of 3 mm-thickness. Experiments were conducted in a reactor which a design allows a homogeneous distribution of magnesium with efficient agitation. An empirical model was used to model the growth kinetics of the diffusion zone by using the Rosin–Rammler equation and estimate particle size distribution. The results were extrapolated to decrepitated magnet powder particles to simulate the neodymium extraction performances. Remarkably, the treatment time required was relatively short, not exceeding 22 minutes, and varied depending on the extraction target and temperature. Both temperature and the setpoint for the volume rate to be treated emerged as critical factors. Their impact on the process was thoroughly examined and discussed. These findings offer promising insights into the industrial feasibility of the use of liquid magnesium for rare-earth extraction from spent permanent magnet.

## Abstract 2

Extraction of rare earth elements from spent NdFeB magnets with liquid magnesium was investigated using a laboratory scale perfect stirred reactor. Data were processed to model Nd extraction performances for treating decrepitated magnet powder.

## Graphical abstract

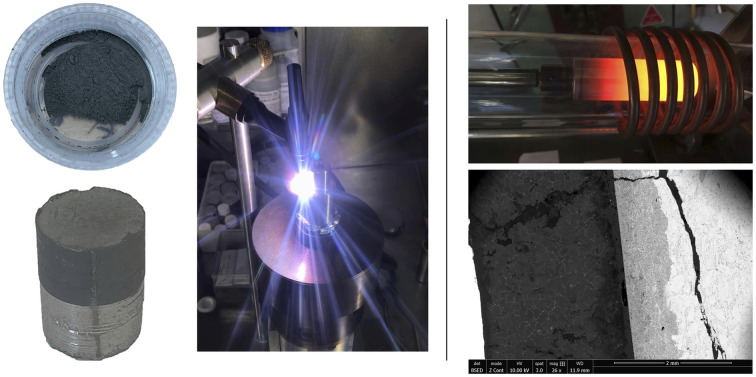

## 1. Introduction

Since their invention in 1983, neodymium–iron–boron (NdFeB) magnets have become key technological components due to their significantly higher stored volumetric magnetic energy compared to ferrite-based (15 times more), samarium-cobalt (2.5 times more) and AlNiCo (7 times more) permanent magnets.^1^ These remarkable properties make them indispensable for producing small and powerful magnets for critical applications such as electronics (hard drives), transportation (hybrid and electric vehicles), and energy (wind turbines), which cover more than half of the world's permanent magnet market.^2^ NdFeB magnets primarily contain 60–70% (wt) iron, 20–25% (wt) neodymium as well as 0–5% (wt) praseodymium, 1% (wt) boron and 0–5% (wt) dysprosium which extends operating temperature range of the magnets up to 200 °C. Besides, traces of other elements (cobalt, nickel and copper) can be found in magnets to improve their performances. A report written by the Joint Research Centre and the European commission's science and knowledge service^3^ indicated that the global cumulative demand for neodymium, praseodymium, terbium and dysprosium was approximately 50 kt in 2018 including 40 kt of neodymium. Another report published by The International Renewable Energy Agency^4^ mentioned that the amount needed for magnet production could grow from 50 kt to 225 kt per year including 180 kt for electric vehicles and 50 kt for wind turbines. In order to mitigate the supply risk (a projected deficit of 135 kt of NdFeB magnets by the end of this decade), numerous projects are focusing on the exploitation of new deposits in Nordic countries, Turkey, Canada and Australia. Furthermore, although magnet recycling alone may not be sufficient to meet magnet production demands. Recycling presents a valuable opportunity to reduce the supply risk of REEs. However, a special attention must be paid to the development of the recycling industry, particularly in Europe where there is almost no mining activity, in Japan and in the United States. Therefore, many governments have recently invested in the rare-earth recycling industry so that NdFeB magnets could contribute as secondary resources for neodymium and dysprosium since recycling could provide up to 2500 tons annum.^5^

**Fig. 1:**
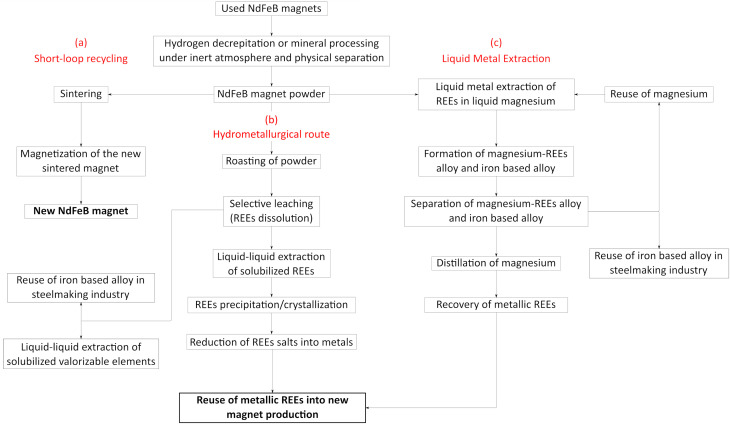
Recycling routes of REEs from NdFeB magnets, (a) short-loop recycling, (b) hydrometallurgical route, (c) liquid metal extraction.

To meet this need and match with the development of the circular economy, promising recycling routes are already under development (Fig. 1). The short-loop recycling route (Fig. 1, route a) appears as one of the most promising approaches. New NdFeB magnets can be produced from spent magnets by hydrogen decrepitation (HD) or other technologies and sintering of the resulting powder.^6^ The HD process (embrittlement of the alloy by hydrogen absorption) is applied to end-of-life sintered magnets in order to produce a powder suitable for further reprocessing to make new magnet by sintering at high temperature and compaction under magnetic field. The main goal in this case is to avoid any degradations of the powder (oxidation is highly detrimental to the magnet properties) and to maintain, as much as possible, the existing microstructure in the powder, in order to recover magnetic performances close to those of pristine magnets. Besides, the HD process can also be applied as a pre-treatment step for further long-loop recycling processes in order to increase their kinetics for extraction of rare-earth elements trough pyrometallurgical and/or hydrometallurgical processes.^7–11^ Hydrogenation Disproportionation Desorption Recombination (HDDR) process is also an extension of the HD process. The HDDR process offers a promising multistage approach to produce anisotropic powders. In the initial stage, it involves the exothermic hydrogenation of both the REE-rich grain boundary phase and the Nd_2_Fe_14_B phase through interstitial absorption of hydrogen. The subsequent stage involves the exothermic disproportionation of the Nd_2_Fe_14_BH_*x*_ phase above 600 °C. After the disproportionation stage, a high-temperature desorption process takes place, during which hydrogen is removed as the reactor is emptied. The final stage encompasses the recombination of the Nd_2_Fe_14_B phase, which is a high-temperature endothermic process.^12–18^

The following reversible reactions describe the HDDR process:

HD processNd_2_Fe_14_B + 2H_2_ → 2NdH_2_ + 12Fe + Fe_2_B

DR process2NdH_2_ + 12Fe + Fe_2_B → Nd_2_Fe_14_B + 2H_2_

More traditionally, the hydrometallurgical route (Fig. 1, route b) allows the recovery of rare-earth elements by combining leaching, solvent extraction, ion exchange and/or precipitation. These techniques implemented for the recovery of REEs from NdFeB magnets are similar to those used in the hydrometallurgical treatment of ores^19^ and are subject of extensive research to increase metal extraction efficiency and mitigate environmental impacts. A thermal oxidative pre-treatment offers selectivity during the leaching step by preventing the dissolution of iron. The decrease of iron concentration in leaching solution is particularly important as the recovery of REEs in the presence of iron is a great challenge in the purification step (liquid–liquid extraction, solid–liquid extraction, precipitation). After liquid–liquid extraction or solid–liquid extraction, precipitation of REEs can be performed to produce sulfate or oxalate salts. These salts must then be converted into metals to make new magnets. As mentioned above, magnets do not contain only neodymium, iron and boron, but also other elements such as praseodymium, dysprosium, nickel, cobalt, copper, *etc.* In some cases, the co-valorization of these metals could be highly beneficial, and iron could be reused in steelmaking industry.

The Liquid Metal Extraction (LME) process (Fig. 1, route c) has been recently investigated to extract REEs from magnets.^20^ LME relies on solid–liquid extraction using a molten metal, primarily magnesium, which acts as a high-temperature solvent due to its strong chemical affinity for REEs. The liquid magnesium extraction process relies on two key steps: (I) diffusion of REEs contained in the magnets by immersion in liquid magnesium and (II) the distillation of magnesium to recover metallic REEs. In a short-loop process, magnesium can be reused in LME after distillation, and iron-based alloy can be reintroduced in the steelmaking industry. Such an operation within a magnet recycling process may reduce effluent generation and environmental impacts. Therefore, LME process may be far less harmful toward the environment than mining as it can be considered as an effluent-free process. The main cost factor of this process depends primarily on magnesium consumption and the energy required to maintain magnesium at a liquid state. The main advantage of this process beside the absence of effluent is the direct production of rare-earth metals, which can be used directly to produce new magnets.

Sparse data on neodymium extraction from magnet into liquid magnesium and growth kinetics of diffusion zone are reported in the literature. However, these data mainly concern experiments conducted between 700 and 800 °C in static systems. In the present work, neodymium extraction in liquid magnesium by contacting spent magnet powder prepared by decrepitation with liquid magnesium was investigated between 700 and 900 °C in a perfectly stirred reactor. These operating conditions ensure a homogeneous chemical composition of the liquid phase and prevent the formation of a neodymium concentration gradient near the magnet interface, where a neodymium-enriched liquid boundary layer could slow the extraction rate.

In this work, the growth kinetics of the diffusion zone characterized by neodymium depletion is investigated. Additionally, time to treat the decrepitated powder using liquid magnesium is estimated aiming to assess the potential for industrial application of liquid magnesium for extracting neodymium from spent NdFeB magnets.

## 2. Materials and methods

Discarded permanent NdFeB magnets from hard disk drives were reduced into powder using HD process at room temperature under a hydrogen pressure of 1 bar. Following hydrogenation, the powder was sieved to isolate particle with sizes below 200 μm (*d*_50_ = 100 μm). Table 1 presents ICP-MS analyses of the resulting decrepitated powder:

**Table 1:** Elemental analyses of the NdFeB magnet powder after decrepitation and sieving

Elements	Fe	Nd	Pr	Dy	B	Others
% (wt)	62.83	23.66	2.90	2.95	1.00	6.66

**Fig. 2:**
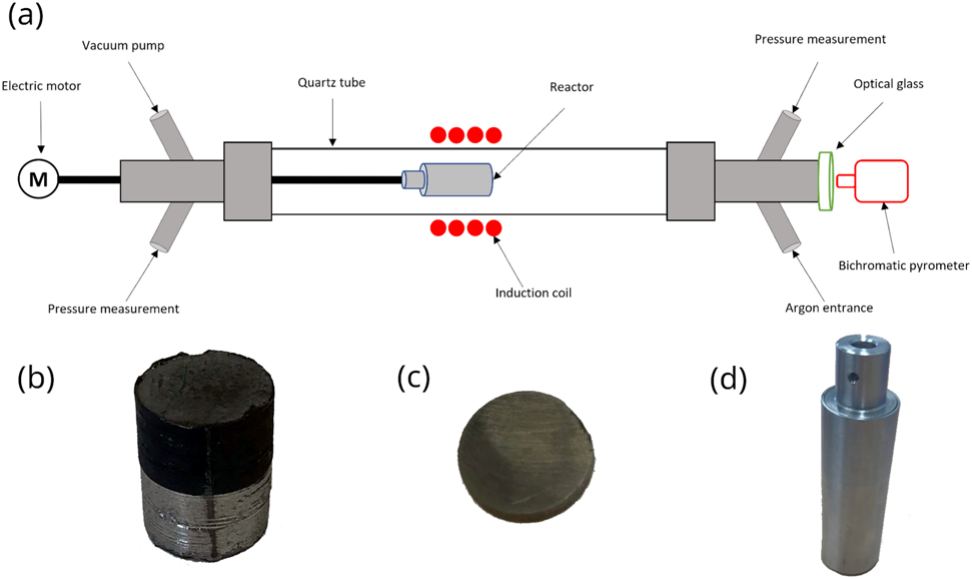
Experimental methodology, (a) heating setup used for rare-earth elements extraction by liquid magnesium, (b) sintered cylinder of magnet powder, (c) 3 mm-thickness cylindrical sample, (d) argon sealed capsule containing magnet powder and magnesium with calcium addition.

Sun *et al.*^21^ studied the extraction mechanisms of neodymium from a magnet between 730 and 930 °C at Mg/NdFeB mass ratio ranging from 0.5 to 2.0. The scraps were initially immersed in an aqueous solution of potassium hydroxide (KOH) for 8 hours to remove oil contamination and smudginess. After washing with water and drying at 180 °C, the magnet was mechanically ground to produce a powder which the particle size was less than 150 μm. Examination of the extraction rate as a function of the treatment time showed 15 minutes of contact between the magnet and liquid magnesium was enough to reach the steady state for neodymium extraction. Therefore, it was expected that neodymium extraction by liquid magnesium may be similar for the decrepitated NdFeB magnet which the particle size was lower than 200 μm. In the present study, it was necessary to slow down the neodymium diffusion to investigate the kinetic growth of the diffusion zone since the steady state was reached too quickly in the experimental setup displayed in Fig. 2a. The neodymium diffusion at the magnet/magnesium interface was slow down by sintering the powder produced by decrepitation. Powder was sintered by filling a carbon graphite mold (15 × 20 mm cylinder) in a hot press for 10 hours at 1000 °C and 40 MPa under argon atmosphere (Fig. 2b). After sintering, the cylinder was cut into slices of 3 mm thickness to investigate neodymium diffusion at the magnet/magnesium interface (Fig. 2c).

Na *et al.*^22^ showed that the extraction efficiency of REEs by liquid magnesium decreased with decreasing the particle size of NdFeB magnet. Neodymium extraction from particle size of 500 μm resulted in the formation of an alloy containing 19% (wt) of neodymium in magnesium after 50 minutes treatment while extraction from particle size of 5 mm resulted in a neodymium content of 24% (wt) under the same experimental conditions. Nam *et al.*^23^ reported the same observation for dysprosium extraction by liquid magnesium from NdFeB magnets in granular media after 6 to 48 hours of treatment. Particle size influenced the extraction efficiency, with 76% (wt) of dysprosium being extracted after 24 hours of treatment when the particle size was below than 200 μm. In contrast, the extraction efficiency reached 81% (wt) when the particle size was greater than 2 mm. Similarly, the extraction efficiency reached 97% (wt) of dysprosium after 48 hours when the particles size was greater than 2 mm while particle size below than 200 μm resulted in 94% (wt) of dysprosium extraction. These authors explained these counterintuitive results by the presence of oxide layers onto the surface of small particles, which negatively affects neodymium and dysprosium extraction.

Akahori *et al.*^24^ conducted a parametric study of the diffusion of neodymium and dysprosium into liquid magnesium at 1000 °C, considering similar behavior between praseodymium and neodymium. In this study, the magnet powder was obtained by grinding NdFeB magnets so that the particle size ranged between 300 and 700 μm. A complete extraction of neodymium was achieved after 6 hours of treatment when the Mg/NdFeB mass ratio was between 2 and 40. A significant improvement in dysprosium extraction was observed in the presence of calcium, which facilitates the reduction of rare-earth oxides into rare-earth metals. Therefore, calcium chips were added to the capsule containing the magnet cylinder and magnesium in order to avoid interference of the oxygen with the diffusion of REEs in the liquid magnesium. In 2017, Park *et al.*^25^ demonstrated that a high Mg/NdFeB mass ratio ranging from 6 to 15 significantly improved magnesium penetration into the magnet leading to a drastic increase in dysprosium extraction efficiency, from 58% (Mg/NdFeB mass ratio = 6) to 66% (Mg/NdFeB mass ratio = 15). They noted that this ratio is less critical for neodymium extraction than for dysprosium extraction, as neodymium was fully extracted at a Mg/NdFeB mass ratio of 7.

Each sample was placed in a stainless-steel capsule measuring 80 × 28 mm (Fig. 2d) along with magnesium at a Mg/NdFeB mass ratio of 5 which was limited by the capsule volume. Calcium chips were also added. The capsule was hermetically sealed by arc welding under an argon atmosphere in a glove box (Jacomex©) with an oxygen content of less than 0.5 ppm.

In this study, the bulk densities (*ρ*_b_) of the samples were measured 6 times at 25 °C using a pycnometer resulting in values of *ρ*_b_ = 7.71, 7.27, 7.44, 7.78, 7.76, 7.77 g cm^−3^. The average value was found to be 7.62 g cm^−3^ with a 90% confidence interval ranging from 7.45 to 7.80 g cm^−3^. According to Sagawa at al., Brown *et al.* and Xia *et al.*, the true density (*ρ*_t_) of NdFeB magnets was 7.54 g cm^−3^ at 25 °C.^26–28^ Using this information, the porosity of the material (*ε*) can be calculated following the following equation:1
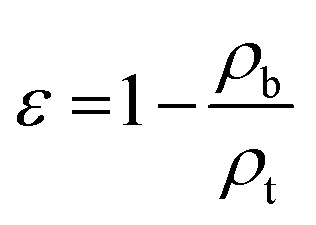


The lowest value within the confidence interval, *i.e. ε* = 1.20%, was used to estimate the material's porosity. This value was considered negligible.

**Fig. 3:**
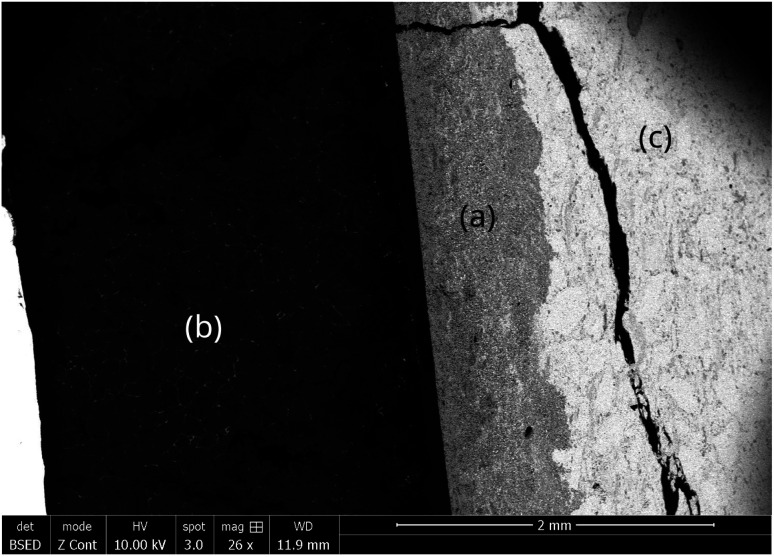
SEM photography of the magnet-magnesium system after extraction at 900 °C for 60 min. (a) Diffusion zone, (b) REEs enriched magnesium zone and (c) zone in which the magnet was not affected by diffusion.

The capsule displayed in Fig. 2d was held inside an induction coil to heat the sample (Fig. 2a). It was attached to a rotating rod driven by an electric motor, which allowed chemical homogenization of the liquid magnesium throughout the treatment and ensured perfectly stirred reactor conditions at 27 rpm. The axis of rotation was inclined at 15° to confine the material at the bottom of the capsule. A quartz tube connected to a vacuum pump (Franklin electric © model 1101006414) and an argon source covered the hot zone to provide inert conditions. A bichromatic pyrometer (Optris©, model OPTCTRF1ML) was used to measure and record the temperature during the experiment. The pyrometer was calibrated by comparing its temperature measurements with the temperatures recorded by two thermocouples located at the side of the capsule and at the vicinity of the location where the pyrometer recorded the temperature. After heating, the magnet was cut into two halves to observe the sample center by scanning electron microscopy after polishing. The samples were polished at 250 rpm on the cut face using silicon carbide grits ranging from 200 μm to 10 μm. Finer polishing was performed using a diamond suspension with particle sizes of 3 μm and 1 μm. Finally, the samples were carefully cleaned in ethanol under ultrasonic agitation for 5 minutes. The concentration profiles in the central zone of the sample where mass transfers occur in a unidirectional manner parallel to the axis of the cylinder were determined by measuring the thickness of the diffusion zone, which is depleted in REEs. These measurements were performed by scanning electron microscopy (Quanta 600). The diffusion zone was identified in each sample by Energy Dispersive Spectrometry (EDS) since the contrast differences observed by EDS was high as shown in Fig. 3. This Figure distinguished the (a) diffusion zone, (b) the solidified magnesium (b) and the inner zone of the magnet (c) unaffected by neodymium extraction from the liquid magnesium.

**Fig. 4:**
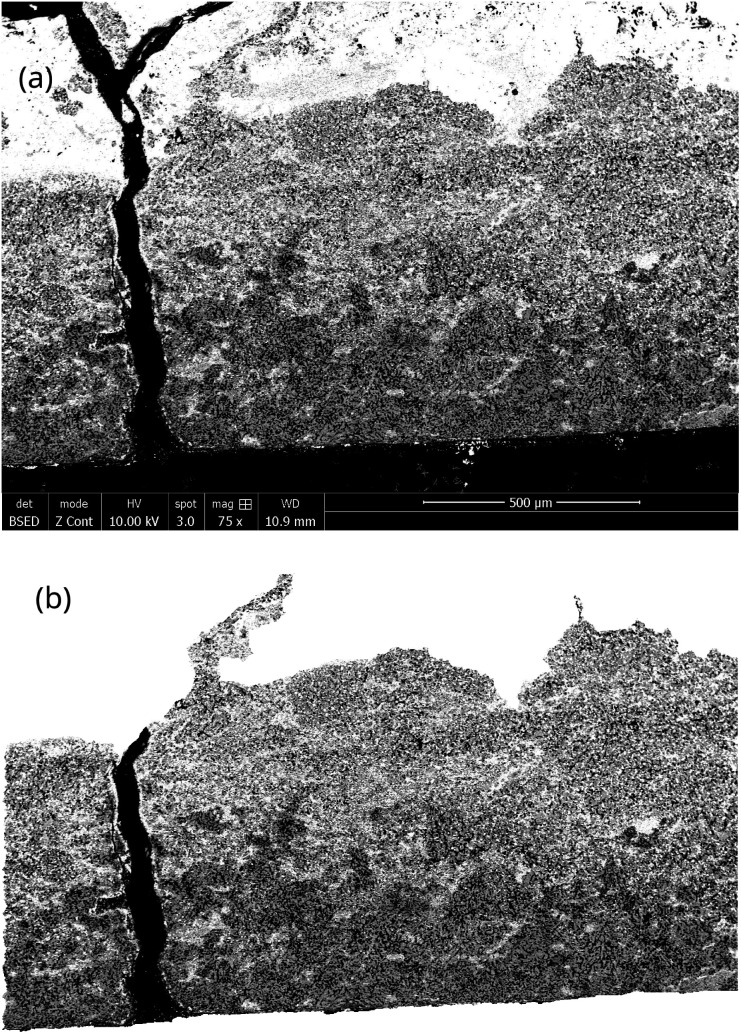
(a) SEM photography of the diffusion zone for image analyses resulting in the identification of (b) the diffusion zone.

SEM images were captured along the entire interface between the magnet and magnesium, covering the total thickness of the diffusion zone through the sample. To analyze the diffusion zones in each image, they were truncated using the contrast image analysis tool in GIMP software (version 2.10.24), as illustrated in Fig. 4. Once the diffusion zone was clearly identified, its thickness was calculated based on the equivalent number of pixels, proportional to the SEM scale used. The average of these estimations from all images taken for the same sample provided the estimation of the diffusion zone thickness.

## 3. Results and discussion

### 3.1 Characterization of the diffusion layer at the magnet-magnesium interface

**Fig. 5:**
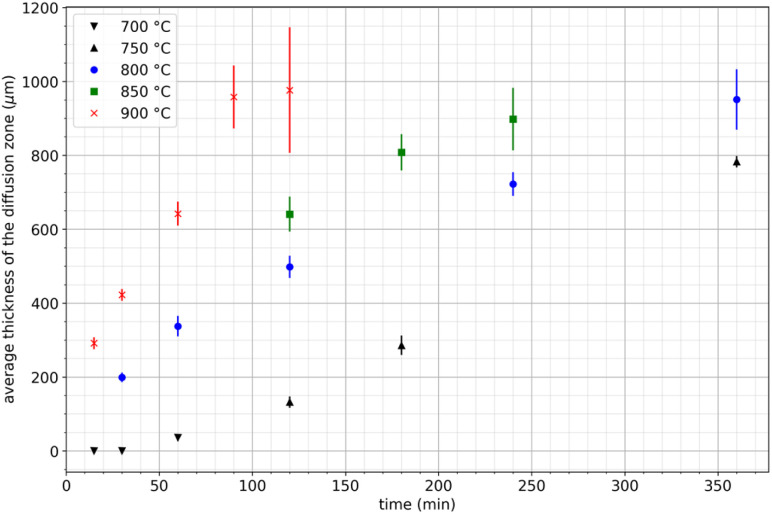
Variation of the average thickness of the diffusion layer during neodymium extraction by liquid magnesium as a function of time between 750 and 900 °C.

For this study, nineteen extraction tests were carried out at temperatures ranging from 700 to 900 °C during 15 to 360 minutes. Table 2 reports the results of all the extraction tests conducted in this study. Fig. 5 shows that the average thickness of the diffusion zone increases with temperature and treatment duration, measured in μm. Thus, REE transport phenomena in the diffusion layer may be the limiting factor of the extraction mechanism. For the lowest temperature (700 °C), the average thickness of the diffusion zone remains constant for approximately 30–60 min before increasing. This phenomenon was consistently observed in all three trials at this temperature. Therefore, it was not attributed to the heating ramp or any bias related to temperature measurement. This incubation time was not observed at the other temperatures, likely because no data were recorded during the incubation time, which may occur very early in the process.

**Table 2:** Average thickness of the diffusion zone as a function of temperature and time treatment

Duration (min)	Temperature (°C)	Average thickness of the diffusion zone (μm)
15	700	0
30		0
60		36.39
120	750	131.98
180		285.6
360		782.78
30	800	199.18
60		337.54
120		498.24
240		722.25
360		950.92
120	850	640.6
180		807.93
240		897.92
15	900	291.56
30		422.41
60		717.02
90		957.80
120		976.43

**Fig. 6:**
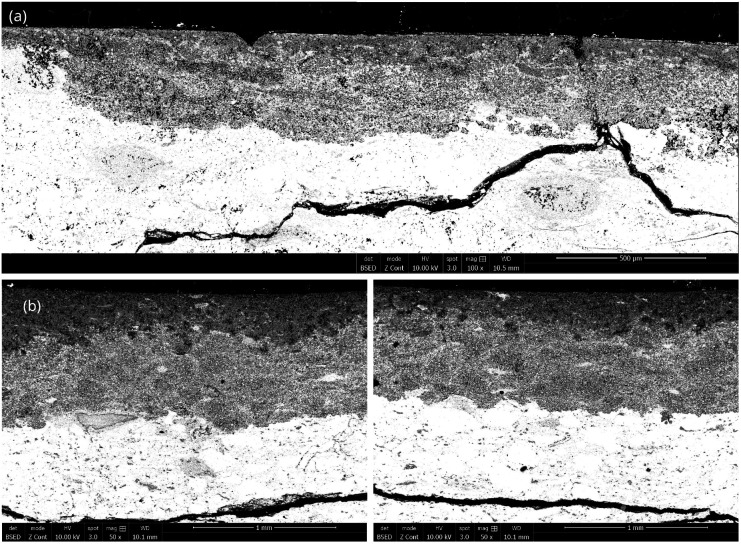
SEM photography of the diffusion layer obtained at (a) 800 °C for 1 hour (magnitude ×100) and (b) 900 °C for 2 hours (magnitude ×50) with different thicknesses.

SEM images revealed significant variations in the characteristics of the diffusion layer, as depicted in Fig. 6 following 1 hour of thermal treatment at 800 °C and 2 hours of thermal treatment at 900 °C. Below 750 °C, samples displayed only a few scattered diffusion layers near the edge in contact with magnesium, with the majority of the material remaining unaffected. Interestingly, the observed diffusion layers at these lower temperatures exhibited uniform thickness, resulting in a narrow confidence interval for a highly precise average thickness measurement. Conversely, at higher temperatures, diffusion layers were observed along the entire interface of the sample but exhibiting irregular thickness. This phenomenon became more pronounced as the thickness exceeded 500 μm and led to a reduction in uniformity particularly evident beyond above 900 μm.

**Fig. 7:**
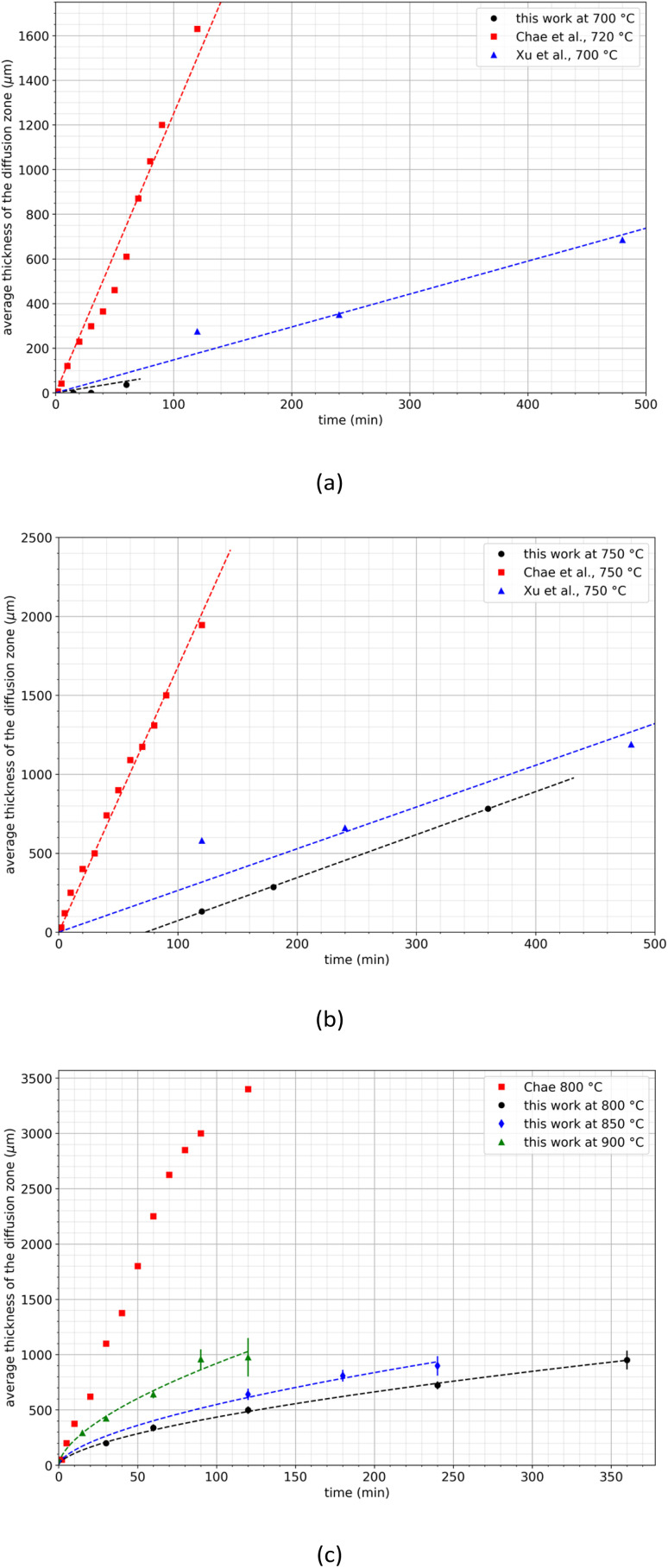
Comparison of the diffusion zone growths at (a) 700–720 C, (b) 750 °C and (c) at temperatures above 800 °C. The dash lines were calculated by using eqn (3).

Xu *et al.*^29^ and Chae *et al.*^30^ studied the diffusion of neodymium into liquid magnesium between 675 and 800 °C. They observed the formation of a REE-depleted zone at the periphery of the samples, and they correlated the thickness of this zone with the temperature and the treatment duration. Fig. 7 compares the experimental data obtained in the present study with those obtained by these authors.

A significant discrepancy is obvious between the studies conducted by Xu *et al.* and Chae *et al.* particularly in terms of the growth rates of the diffusion layer. For example, at 700 °C, Xu *et al.* reported a growth rate of 1.5 μm min^−1^ while Chae *et al.* observed a much high rate of 12.5 μm min^−1^. Similarly, at 750 °C, Xu *et al.* recorded a growth rate of 2.6 μm min^−1^ while Chae *et al.* reported a considerably faster rate of 16.8 μm min^−1^. Notably, Chae *et al.* did not observe any incubation time whereas it was not possible to draw a conclusion regarding the work of Xu *et al.* since they did not record data at the earliest time points. Furthermore, above 800 °C, Chae *et al.* noted a linear increase of the thickness of the diffusion zone up to 2700 μm. Beyond this temperature, the thickness increase was no longer linear, indicating a decrease in the growth kinetics of the diffusion layer (Fig. 7c).

This observation is in fair agreement with the results obtained in the present work even if the values of the thickness of the diffusion zone reported by Chae *et al.* are higher. The diffusion layer growth remains significantly lower in the present work than that obtained by Chae at 800 °C and even at 900 °C. This significant discrepancy between the present results and those reported in the literature may be explained by differences in the preparation of the sintered magnets used in this work. SEM observations revealed the presence of magnesium in the diffusion layer as it was found by Xu *et al.* and Chae *et al.* This can be due to diffusion, but also because of magnesium infiltration, the latter being particularly influenced by the magnet porosity. As mentioned above, the magnet powder was sintered under severe conditions to achieve negligible porosity in the massive sample. As a consequence, mechanisms of magnesium infiltration are drastically limited, allowing to observe on a large scale the extraction kinetics that would be obtained at the powder scale. Finally, this approach allows to extrapolate results to the decrepitated powder particle size scale. The following empirical law can be used to describe the evolution of the thickness of the diffusion zone as a function of time and temperature at temperatures greater than 800 °C as shown in Fig. 7:

2 *l*(*t*,*T*) = *A*(*T*)*t*^0.61^

Here, *A*(*T*) represents an empirical parameter, with specific values assigned at different temperatures: (*A*(800 °C) = 26.2 μm min^−0.61^, *A*(850 °C) = 33.0 μm min^−0.61^ and *A*(900 °C) = 55.4 μm min^−0.61^. While a comprehensive explanation of the underlying mass transfer phenomena will necessitate further investigation to develop a more detailed model for the kinetics of diffusion layer growth, this initial approach serves as a valuable foundation for conducting preliminary calculations related to the treatment duration required for REEs extraction from decrepitated magnet powder, particularly from an industrial perspective.

### 3.2 Estimation of the duration of the neodymium extraction by liquid magnesium

**Fig. 8:**
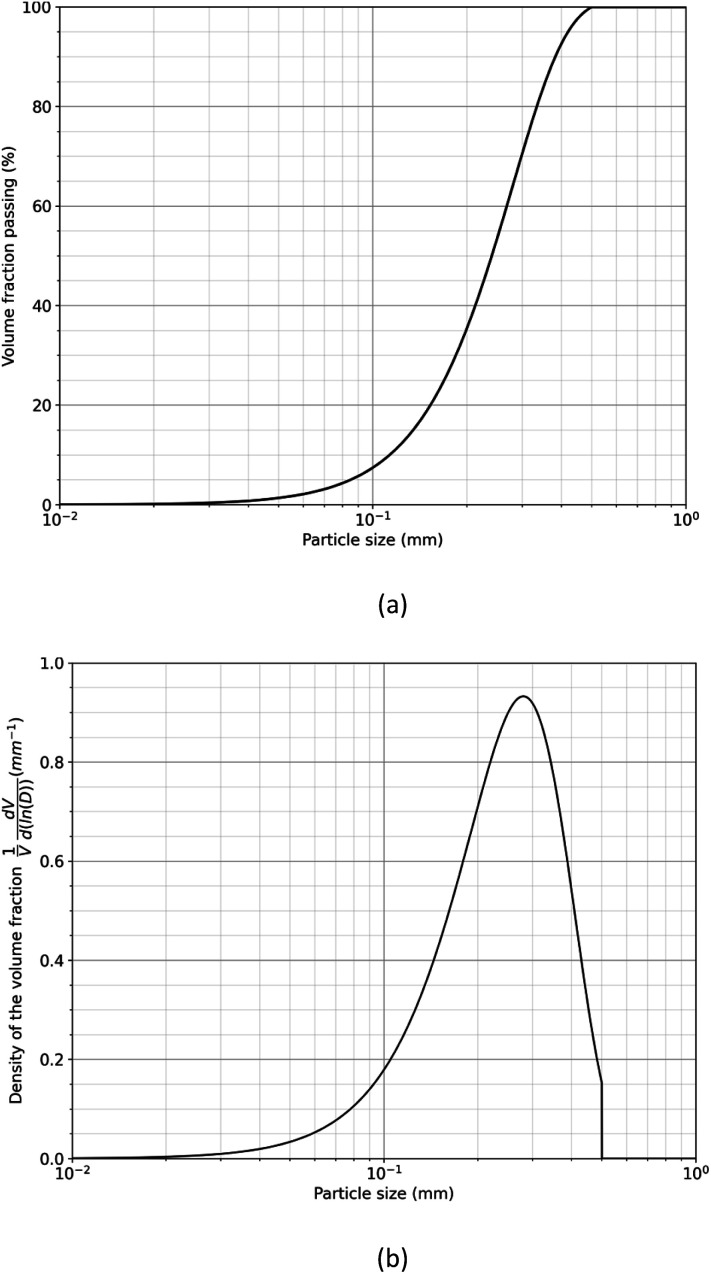
(a) Volume fraction passing and (b) density of the volume fraction as a function of particle size distribution of powder produced from decrepitated magnet calculated by the Rosin–Rammler equation.

The duration required to fully treat magnet particle produced by HD with liquid magnesium is estimated by considering the growth of the diffusion layer at the interface between the magnet and liquid magnesium. To facilitate this estimation, the particle size distribution of the decrepitated powder was modelled using the Rosin–Rammler equation,^31^ assuming a sieve size of 500 μm:3
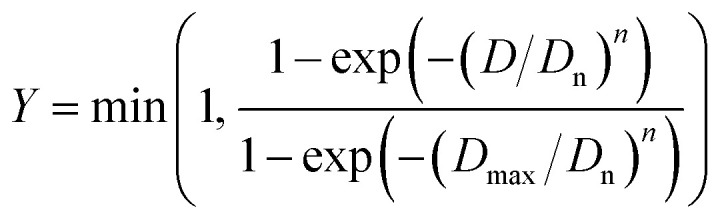
Here, *Y* represents the fraction of size smaller than the diameter *D*, *D*_n_ is the characteristic diameter of the particle population, *D*_max_ is the maximum diameter of the particles, and *n* denotes for the dispersion of the particle size distribution. According to Rivoirard *et al.*,^6^ the particle size distribution can reasonably be described by the Rosin–Rammler equation by considering *D*_n_ = 3.5 10^−4^ m, *D*_max_ = 5 10^−4^ m and *n* = 2.5. Fig. 8a illustrates the volume fraction passing and Fig. 8b the density of the volume fraction as a function of particle size distribution calculated by using eqn (3).

By using the estimated growth rate of the diffusion layer, the volume fraction of treated powder can be expressed as a function of temperature and time. For this purpose, it was assumed that each grain was a sphere of diameter *D* and that neodymium extraction by liquid magnesium was responsible for the formation of a diffusion zone *l*(*T*,*t*) in the treated volume fraction (*x*_treated_) of such a grain defined by:

4 If *D* < 2 *l*(*T*,*t*) then *x*_treated_ = 1

**Fig. 9:**
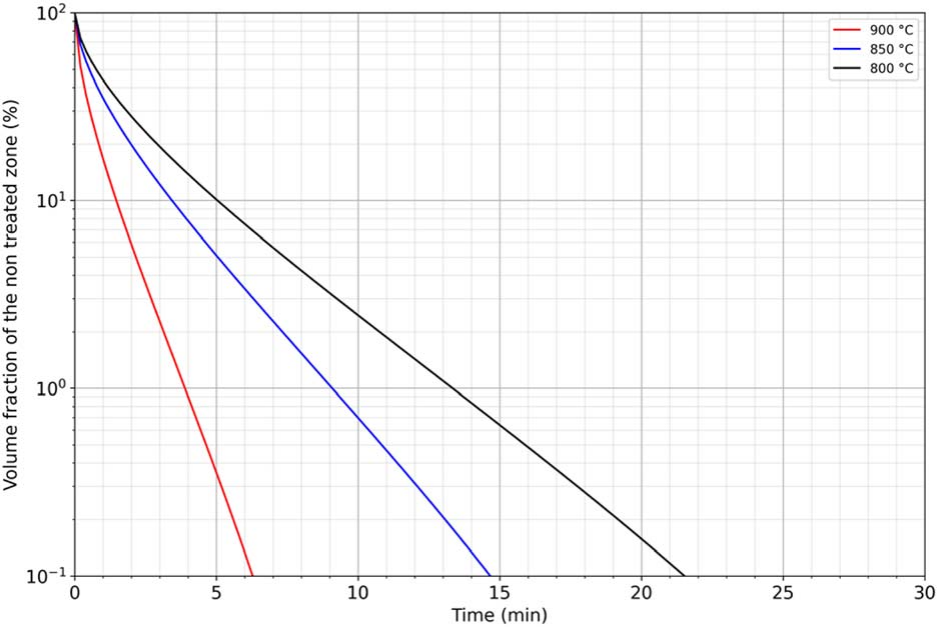
Modelling of the volume fraction of residual untreated powder as a function of temperature and immersion time in liquid magnesium.

5
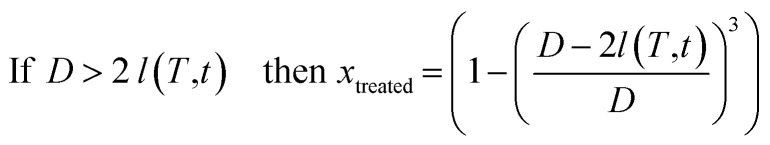
From eqn (4) and (5), it is thus possible to calculate the remaining volume fraction of the untreated powder (*x*_untreated_):
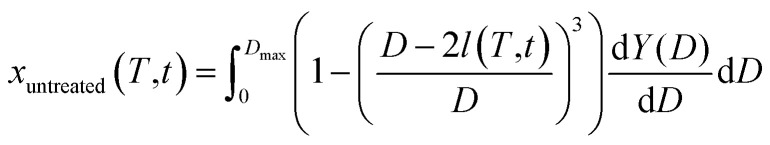


Fig. 9 shows the volume of residual untreated powder as a function of temperature and extraction time. The treatment can be relatively short to extract neodymium by liquid magnesium since less than 22 minutes is enough whatever the temperature as it was reported by Na *et al.* Temperature represents a critical parameter on the treatment duration. A decrease of 100 °C, from 900 °C to 800 °C, results in an increase of the treatment duration of 250%, from 6 to 22 minutes, to reach 99.9% of treated powder.

In addition, the target fraction to be treated influences strongly the treatment duration. For example, the treated volume fraction can reach 90% of the material if the treatment duration lasts between 1.5 and 5 minutes depending on the temperature, while a target of 99% would multiply the treatment duration by 2.5.

These preliminary calculations show that this extraction method could be considered for industrial use to extract REEs from decrepitated powders. Complementary studies could also be carried out at higher temperatures. Nevertheless, performances obtained at 900 °C are presently highly attractive and could stand for an optimum range of temperature for such a process treatment.

## 4. Conclusions

In this paper, a laboratory-scale equipment specifically designed for liquid magnesium extraction (LME) of REEs from used NdFeB magnets decrepitated under hydrogen has been presented. The series of experiments conducted between 700 and 900 °C for time treatment ranging from 30 minutes to 6 hours enabled the characterization of the growth kinetics of the REEs-depleted zone at the magnet-magnesium interface. These treatments were performed on massive samples sintered from a decrepitated powder under rigorous operating conditions to eliminate internal porosities. The absence of porosity prevents the infiltration of liquid magnesium. Under these conditions, the kinetic laws at macroscopic scale can be used to simulate the neodymium extraction by liquid magnesium on decrepitated powder. High consistency with the work of Xu *et al.* was obtained while high discrepancies were observed when compared to the data reported by Chae *et al.* Such discrepancies may be explained by the difference of porosity between the materials used in the present work and the material used in the work of Chae *et al.* The results provide important insights for the determination of the treated volume fraction and give preliminary indications of the industrial performance of REEs extraction from spent magnets by liquid magnesium. This work used an empirical model describing the froth kinetic of the diffusion layer of neodymium combined with the Rosin–Rammler equation to estimate the optimal duration of liquid magnesium extraction to extract neodymium from decrepitated magnet. Different authors cited previously (Xu *et al.* 2000, Chae *et al.* 2014, Sun *et al.* 2015, Akahori *et al.* 2017) were able to analyse the phases present in the magnet-magnesium system. The main phase present in the magnet is Nd_2_Fe_14_B with Nd_2_Fe_17_, Nd-rich phase at the grain boundaries and α-Fe phase in minor presence. In the zone affected by the diffusion of REEs into magnesium, phases are the same but the α-Fe is major this time and many Nd-rich phases are very present at the edge with Mg. Finally, the alloy formed is mainly α-Fe with low percentage of Nd (2–8%) surrounded by Mg_12_Nd phase (30–37% Nd) at the grain boundaries. However, no mechanisms or relation between kinetics and extraction efficiency have been reported yet. Moreover, Nam *et al.*^32^ performed a detailed study of dysprosium extraction by liquid magnesium and showed that heat treatment led to the formation of the intermetallic compound Dy_2_Fe_17_, which may diffuse less rapidly into the magnesium. They also showed that dysprosium oxides may diffuse even more slowly, and that elemental diffusion decreases according to the following order: Dy_2_Fe_14_B > Dy_2_Fe_17_ > dysprosium oxides. Given that neodymium is not significantly different chemically from dysprosium, it is reasonable to assume that neodymium compounds exhibit a similar behaviour, albeit likely in a shorter time frame. In conclusion, further research is required to deepen the understanding of the extraction mechanisms. A comprehensive characterization using an electron probe micro-analyser could facilitate more precise concentration measurements of REEs and magnesium in the diffusion layer, offering a more detailed description of the mass transfer phenomena occurring in this zone.

## Author contributions

Conceptualization: T. Q., A. C.; formal analysis: T. Q., A. C., N. S., J. M.; funding acquisition: A. C.; investigation: N. S., J. J.; project administration: A. C., T. Q.; supervision: T. Q., A. C.; visualization: N. C.; writing-original draft: N. C., T. Q.; writing-review and editing: A. C., T. Q.

## Conflicts of interest

The author declares no conflict of interest.

## Supplementary Material
